# Effects of Dexmedetomidine on Immune Cells: A Narrative Review

**DOI:** 10.3389/fphar.2022.829951

**Published:** 2022-05-02

**Authors:** Rui Chen, Yan Sun, Jing Lv, Xiaoke Dou, Maosha Dai, Shujun Sun, Yun Lin

**Affiliations:** ^1^ Institute of Anesthesia and Critical Care Medicine, Union Hospital, Tongji Medical College, Huazhong University of Science and Technology, Wuhan, China; ^2^ Department of Anesthesiology, Union Hospital, Tongji Medical College, Huazhong University of Science and Technology, Wuhan, China

**Keywords:** dexmedetomidine, immune cells, innate immune response, adaptive immune response, inflammatory factors

## Abstract

As we all know, dexmedetomidine (DEX), as a highly selective α_2_ adrenergic receptor agonist, exerts sedative, anti-anxiety and hypnotic effects by inhibiting the discharge of norepinephrine neurons in locus coeruleus and GABA-related hypnotic pathways. However, the role of DEX in anti-inflammatory and immune regulation has gradually attracted the attention of researchers in recent years. The α_2_ adrenergic receptor is one of the members of the adrenergic receptor family, which is widely present in a variety of immune cells and mediates the biological behavior of the inflammatory immune system. At present, there have been more and more studies on the effects of DEX on immune cells and inflammatory responses, but few studies have systematically explored the anti-inflammatory and immunomodulatory effects of DEX. Here, we comprehensively review the published human and animal studies related to DEX, summarize the effects of DEX on immune cells and its role in related diseases, and propose potential research direction.

## 1 Introduction

The adrenergic system is closely linked to the immune system (Keating, 2015). Both innate immune cells and adaptive immune cells express adrenergic receptors and can directly respond to the sympathetic nervous system (Lorton and Bellinger Denise, 2015; Scanzano and Marco, 2015). Primary and secondary lymphoid tissues are regulated by postganglionic sympathetic nerve fibers, mainly secreting norepinephrine as its main neurotransmitter (Sharma and Farrar, 2020). In general, the role of the adrenergic system in immunity is gradually receiving attention and has now become a research hotspot.

Dexmedetomidine (DEX) is a highly selective α_2_ adrenergic receptor (AR) agonist, which has a high affinity for the AR family members α_2_A and α_2_C (Berkowitz et al., 1994; Huang et al., 2021). It can regulate the release of norepinephrine by activating the α_2_ receptor located on the presynaptic membrane, which is the basis of its immunomodulatory function. Meanwhile, studies have shown that DEX can regulate cellular immunity, suppress the inflammatory response in the tissues and enhance the immune function of patients (Li et al., 2016; Ferreira and Bissell Brittany, 2018). However, the effects of DEX on immune cells and inflammatory cytokines have not been systematically summarized.

The main purpose of our review is to comprehensively provide the latest evidence on the immune regulation and anti-inflammatory effects of DEX in various immune cells and immune-related diseases. And the future value of DEX in the treatment of some common but troublesome diseases is worth looking forward to.

## 2 DEX's Regulatory Effect on Immune Cells and Inflammation

### 2.1 General Effects

DEX can not only modulate the ability of antigen-presenting cells to uptake and process antigens, and the recruitment, chemotaxis and local aggregation of immune cells in innate immunity, but also regulate the CD4+/CD8+ ratio and balance the quantity of Th1, Th2, Th17 and Regulatory T cells (Tregs) in the adaptive immunity. As well, DEX can reduce the secretion of pro-inflammatory cytokines (IL-1β, IL-6, IL-8, IL-12/23 (p40), IL-17A, IL-18, IFN-γ, TNF-α, Eotaxin, HMGB1, MIP-2, MCP-1) and increase the level of anti-inflammatory factors (IL-2, IL-4, IL-10 and TGF-β1). Therefore, it plays a vital role in immunity and inflammation.

In more details, here below we summarize the main effects of DEX on different immune cell populations, as shown in Table1.

**TABLE 1:** Summary table on effects of DEX on immune cells and inflammatory cytokines.

Target cells	Effects	
	Suppression	Induction	References
Dendritic cells	Pro-inflammatory cytokines (TNF-α, IL-1β IL-6, IFN-γ)	Anti-inflammatory cytokine IL-10	Ueshima et al. (2013)
	Immunomodulatory factor (IL-12, IL-23)		Chen G. et al. (2016)
	Class II MHC and costimulatory molecules (I-Ab And CD86)		Guo et al. (2018)
			Huang et al. (2021)
Natural killer Cells	Development and metastasis of tumor	Increase the number and maintain the activity	Zhao et al. (2013)
			Yang et al. (2017)
			Wu L. et al. (2015)
Eosinophils	Chemokines (eotaxin)	------------------------	Kalbach et al. (2019)
Mast cells	Degranulation Proteolytic enzyme MMP-9	Proteolytic enzyme MMP-2	Tüfek et al. (2013)
			Matsumoto (2009)
Neutrophils	Pro-inflammatory cytokines (IL-6, TNF-α, Necrosis factor)	Elimination of pathogen	Chen S. L. et al. (2016)
	Antimicrobial effectors (ROS, RNS, NO, iNOS) Respiratory eruption		Yuan et al. (2020)
	Local aggregation of neutrophils		Cowburn et al. (2008)
Monocytes	The ratio of CD42+/CD14+ Pro-inflammatory cytokines (IL-6, TNF-α)	The ratio of HLADR+/CD14+	Zhou et al. (2017)
	The expression of Cx43, PKC-α, VLA-4 and LFA-1		Chai et al. (2020)
	Monocyte-endothelial cells adhesion		
Macrophages	Pro-inflammatory cytokines (IL-6, COX-II, PGE2, HMGB1)	TNF-α, IL-1β Transforming growth factor TGF-β1	Martinez Fernando and Gordon (2014)
	Inflammatory protein MIP-2	Anti-inflammatory cytokine (IL-10)	Lai et al. (2009)
		The production of Th1 cells by promoting the secretion of IL-12 Polarization of M2	Zhou et al. (2020)
		Clearance of Neutrophil and autophagy of mitochondrial	Li et al. (2021)
			Wang K. et al. (2019)
			Piazza et al. (2016)
B cells		Chemokine (IL-2)	Ma et al. (2017)
			Liu et al. (2018)
T cells	The amount of CD8+ The amount of CD3+, CD4+, CD4+/CD8+	Pro-inflammatory cytokines (IL-17A) Immune regulatory factors (IFN-γ)	Huang et al. (2021)
			Yang et al. (2017)
			Lee et al. (2018)
			Song et al. (2020)

### 2.2 Innate Immune Response

The innate immune response refers to the use of differentiated leukocytes to identify and eliminate foreign substances in organs, tissues, blood and lymph. And, the innate immune response-related cells mainly include dendritic cells (DCs), natural killer cells (NKs), eosinophils, mast cells and phagocytic cells (neutrophils and monocytes/macrophages).

#### 2.2.1 Dendritic Cells (DCs)

DCs are one of the antigen presenting cells in the body. They can efficiently capture, process and present antigens, and induce the generation of specific cytotoxic T lymphocytes (CTLs).

DCs express α_1_, α_2_ and β-AR on the cell membrane. As an α_2_-AR agonist, DEX can inhibit immune responses by inhibiting antigen processing/presentation and migration of DCs (Ueshima et al., 2013). For another, DEX could inhibit the maturation and function of DCs by interfering with the synthesis and secretion of IL-12 and IL-23, thereby negatively regulating human immune function (Chen G. et al., 2016). In addition, DEX can inhibit the protein hydrolysis and migration of phagosomes in DCs, reduce the expression and migration of class II MHC molecule I-Ab and costimulatory molecule CD86 and inhibit the proliferation of cytotoxic T lymphocytes and the secretion of IFN-γ, thereby exerting an immunosuppressive effect (Ueshima et al., 2013; Chen S. L. et al., 2016). However, the interesting phenomena have been found in an animal experiments, in which DEX at different concentrations may have the opposite effect on the secretion of inflammatory mediators by DCs. At high concentrations (10, 1, and 0.1 μm) of DEX, the expression of mRNA and the contents of TNF-α, IL-1β, IL-6, and IL-10 in DCs stimulated by LPS increased, while DEX at lower concentrations (0.001 μm), the content of these molecules decreased, and the mechanism was related to the activation of NF-κB and JNK-MAPK signaling pathways (Guo et al., 2018). The experiment shows that DEX has a dual regulatory effect on DCs, in which inflammatory factors are increased at high concentrations and decreased at low concentrations. Studies have found that DEX can preserve the number of DCs in patients undergoing oral cancer surgery and enhance the immune function of patients (Huang et al., 2021).

In general, DEX acts on DCs through α_2_-AR and exerts immune regulation. Guo et al. (2018) have suggested that DEX may play different roles in different pathological conditions, because other kinds of AR and downstream signals also participate in the reaction. Moreover, the current results of clinical trials are too few, and the actual effects of DEX on DCs need to be further confirmed.

#### 2.2.2 Natural Killer Cells (NKs)

NKs are the important immune cells in the body, participating in non-specific cell-mediated anti-tumor immune regulation. They can kill MHC class I cells without being activated, and when the number of NKs decreases, it means the body’s immune function is suppressed (Pilla et al., 2005).

Whether α_2_ -AR are expressed on the cell membrane of NKs is unclear. At present, animal studies have found that DEX can maintain the activity of NKs after surgery or general anesthesia (Taniguchi et al., 2004), which decreased after operation or anesthesia originally. In clinical trials, cancer patients who used DEX in the perioperative period showed significantly higher concentrations of NKs from 6 to 24 h after surgery (Barbera-Guillem et al., 2000; Wolf et al., 2003; Zhao et al., 2013; Yang et al., 2017). It has also been found that the decrease in the number of NK cells after DEX treatment in children with brain tumors during the perioperative period was significantly less than that in the control group (Wu Lei et al., 2015).

In fact, it is believed that IL-2, IL-12, IL-18, IFN-α, TNF-α and leucomodulin (LR) have a positive regulatory effect on the activation and differentiation of NK cells, which can significantly improve the killing activity of NK cells. Prostaglandins (PGE1, E2, D2) and adrenocortical hormone can inhibit the activity of NK cells (Alboni et al., 2010; Kallioinen et al., 2019).

In conclusion, DEX can increase the number of NKs and maintain their activity. However, most of the current studies focus on the effect of DEX on the number of NKs, and lack of studies on the effect of DEX on the differentiation and migration of NK cells. Whether DEX can affect the function of NK cells through the above-mentioned inflammatory immune molecules will be the direction of future research.

#### 2.2.3 Eosinophils

Eosinophils have the function of killing bacteria and parasites, which are also considered as the crucial cells in the process of immune and allergic reactions.

It has not been verified that whether α_2_-AR are expressed on the cell membrane of eosinophils cells yet. But, Kallioinen et al. (2019) and Dahl. (1991) confirmed that eosinophil chemoattractant factor (eotaxin) decreased significantly in healthy subjects after administration of DEX. Eotaxin is a potent chemotactic agent for eosinophils, which mediates leukocyte recruitment in allergic diseases such as asthma (Cheng et al., 2002), and is also strongly up-regulated in septic mouse models (Kalbach et al., 2019). It means that DEX may be a good choice for anesthesia for patients with asthma and sepsis. However, some studies have found that a large proportion of children with severe asthma require an upgrade from noninvasive positive pressure ventilation to invasive mechanical ventilation when DEX is given as adjunctive therapy (Kalbach et al., 2019).

Generally speaking, there are few studies on the effect of DEX on eosinophils currently, and the clinical effect of DEX on asthma can be used as a future research direction.

#### 2.2.4 Mast Cells

Mast cells are the first cells to be recruited to the site of the injury, then selectively produce pro-inflammatory mediators, thereby enlisting neutrophils, macrophages and other monocytes into the site to activate the inflammatory response (Kennelly et al., 2011; Younan et al., 2011; Theoharides et al., 2012).

It is not known that whether α_2_ -AR are expressed on the cell membrane of mast cells. However, early studies have shown that α_2_-AR agonist clonidine can regulate the function of mast cells through α_2_-AR (Lindgren et al., 1987; Anderson et al., 1987; Lavand’homme and Eisenach, 2003; Lavand’homme Patricia et al., 2002). Compared with clonidine, DEX has 8-fold higher affinity for α_2_-AR (Bhana et al., 2000). At present, numerous literatures have shown that DEX may be a strong stabilizer of mast cells, and may inhibit inflammation by preventing degranulation (Tüfek et al., 2013). In the animal models, the application of DEX could reduce oxidative stress (Tüfek et al., 2013). Specifically, it decreased the levels of matrix metalloproteinase-9 (MMP-9) and galectin-3, and increased the level of matrix metalloproteinase-2 (MMP-2) (Matsumoto, 2009). Besides, some studies have found that DEX stabilizes mast cells at the injured site (Tüfek et al., 2013).

There are few studies on the regulation of mast cell function and inhibition of inflammation by DEX, which may be the fields of future research.

#### 2.2.5 Neutrophils

Neutrophils, also known as polymorphonuclear leukocytes (PMN), are the main cell type of the innate immune system, which mainly scavenge pathogens and lead to the acute inflammation (Cowburn et al., 2008). The clearance of pathogens by neutrophils involves a series of physiological processes, including chemotaxis, phagocytosis and killing microorganisms (Kantari et al., 2008; Raffaghello et al., 2008).

It has been identified that α_2_-AR are expressed on the cell membrane of neutrophils (Panosian and Marinetti, 1983). And it has been confirmed that a variety of anesthetics, including propofol, midazolam and ketamine, can inhibit the chemotaxis and phagocytosis of neutrophils and the production of superoxide anion, while DEX not (Stevenson et al., 1990; Mikawa et al., 1998; Nishina et al., 1998). This suggests that DEX may be more suitable for patients with infection, sepsis and systemic inflammation (Nishina et al., 1999).

Whether neutrophils can successfully eliminate pathogens depends on oxidative burst, the main process that kills microorganisms through the formation of reactive oxygen species (ROS) and reactive nitrogen species (RNS) (Cowburn et al., 2008). It was found that DEX could inhibit the oxidative burst stimulated by *E. coli* and the production of nitric oxide (NO) (Chen G. et al., 2016). And studies have found that DEX can inhibit endotoxin induced inflammatory response, reduce the concentration of IL-6, TNF-α and local aggregation of neutrophils, so as to play the role of anti-inflammation and immunosuppression. And the inhibition of DEX on the infiltration of neutrophils may be related to FOXO3a signaling pathway (Yuan et al., 2020).

To sum up, DEX can not only regulate the chemotaxis and phagocytosis of neutrophils, but also inhibit the local aggregation, oxidative burst and the production of reactive oxygen and reactive nitrogen of neutrophils, thereby playing an anti-inflammatory effect. However, the above results are all *in vitro* data, and the clinical situation cannot be simply inferred. Therefore, further studies are needed to elucidate the effect of DEX on function of neutrophils *in vivo*.

#### 2.2.6 Monocytes/Macrophages

##### 2.2.6.1 Monocytes

Monocytes are the precursors of macrophages and DCs, which are involved in immune responses. After phagocytosis of antigen by monocytes, antigenic determinants are transferred to lymphocytes to induce lymphocyte-specific immune responses. And the recruitment of circulating monocytes to inflammatory tissues is one of the important characteristics of acute and chronic inflammatory response.

It has been confirmed that α_2_-AR are expressed on the cell membrane of monocytes. And it has been reported that the percentage of monocyte-platelet aggregation (CD42a +/CD14 +) can reflect the level of inflammation and hemostasis, and the percentage of monocyte activated cytokines (HLA DR+/CD14^+^) can reflect the state of immunosuppression (Monneret et al., 2006). Clinical trials have found that when DEX acts on monocytes, it can inhibit the inflammatory response and enhance immunity by inhibiting the percentage of (CD42a+/CD14^+^), promoting the percentage of (HLA DR+/CD14^+^) and reducing the production of proinflammatory cytokines, such as IL-6 and TNF-a (Zhou et al., 2017).

*In vitro* cell experiments, DEX decreased Cx43 expression and PKC-α of the carboxyl terminal domain of Cx43 protein in monocytes at its clinically relevant concentrations (0.1 and 1 nm). With the downregulation of PKC-α, the NOX2/ROS signaling pathway was inhibited, resulting in the decreased expression of VLA-4 and LFA-1, and finally, decreased monocyte-endothelial cell adhesion (Chai et al., 2020).

At present, there are many known risk factors that increase monocyte-endothelial cell adhesion, including patients undergoing major surgery, intensive care, or long-term bed rest (Sikorski et al., 2011; Ribeiro et al., 2018; Schmitt et al., 2020; Wilhelms et al., 2020; Wu et al., 2020). Therefore, the rational use of DEX in critically ill patients should be concerned. Moreover, whether DEX can affect the phagocytosis and presentation of monocytes remains to be further studied.

##### 2.2.6.2 Macrophages

Macrophages, as the principal phagocytes in the inflammatory stage, are responsible for clearing the necrotic fragments, pathogens of tissues and cells in the body damage. They can phagocytize and absorb polymorphonuclear neutrophils (PMN) with local infiltration and apoptosis, and then inhibit the excessive secretion of pro-inflammatory cytokines. They play a key role in the lysis phase, and participate in the progress and regression of inflammation (Sugimoto et al., 2017).

Macrophages express α_2_-AR on their cell membrane. And the ability of macrophages to resist microbial growth depends on the activated state of macrophages. There are two phenotypes of macrophage activation including M1 and M2. M1 macrophages participate in the positive immune response and play the role of immune monitoring by secreting pro-inflammatory cytokines and chemokines, and presenting antigens; M2 macrophages only have weak antigen presenting ability, but play an important role in immune regulation by secreting cytokines such as IL-10 and TGF-β (Martinez Fernando and Gordon, 2014). And DEX can enhance the production of major antibacterial effector molecules, including ROS and NO, and activate macrophages to resist the growth of intracellular pathogens (Rezai, 1968; Ohmori and Hamilton, 1994; MacMicking et al., 1997). It can also activate the antifungal and antibacterial activities of macrophages by combining with macrophages α_2_-AR, and regulate the up-regulation of inflammatory molecules induced by endotoxin (Miles et al., 1996; Lai et al., 2009). In an animal experiment, macrophages were pretreated with DEX to increase pro-inflammatory factors, such as TNF-α and IL-6, inhibit the secretion of anti-inflammatory factor IL-10, and promote macrophage M2 polarization in a PPARγ/STAT3 dependent manner (Zhou et al., 2020).

Moreover, Chang et al. (2013) found that DEX could inhibit the transport of HMGB1 from nucleus to cytoplasm and the expression of high mobility group box 1 (HMGB1) mRNA, while HMGB1 is a key pro-inflammatory factor closely related to the mortality of patients with sepsis. Its mechanism may be related to NF-κB signaling pathway and α_2_-AR activation (Chang et al., 2013). Li et al. (2021) found that DEX post-treatment, through the increase of F4/80 + Ly6G + macrophages, promotes the secretion of TGF-β1, which leads to the reduction of cytokine storm and accelerates the resolution of inflammation. A series of studies have found that DEX can reduce the secretion of IL-1β and TNF-α in macrophages, increase the expression of LC3-II (autophagy related protein), promote the clearance of damaged mitochondria. The DEX also can promote PTEN-induced putative kinase 1 (PINK1) mediated mitochondrial autophagy, thereby reducing the apoptosis and inflammation of macrophages induced by LPS, and play a protective role in sepsis (Wang K. et al., 2019). Yang et al. (2008) found in animal models that high-dose but not clinically relevant dose DEX can effectively inhibit the concentration of macrophage inflammatory protein 2 (MIP-2), TNF-α and iNOS in the lung, and significantly reduce the cytokines (IL-1β, IL-6) in ventilator-related lung injury, these effects are at least partially mediated by α_2_-AR. However, Lai et al. (2009) discovered that DEX at a higher dose than routinely used in clinics has a significant biphasic effect (first inhibition and then enhancement) on the secretion of inflammatory factors (COX-2, PGE2, TNF-a, IL-1b, IL-6, IL-10, iNOS and NO) after activating mouse macrophages α_2_-AR. Meanwhile, it has been found that DEX does not directly inhibit the release of cytokines from human pulmonary macrophages like rodents (Kang et al., 2003; Piazza et al., 2016).

At present, the effect of DEX on phagocytosis of macrophages is still controversial. Wu R. S. et al. (2015) found that DEX could enhance the phagocytic activity of macrophages in mice with endotoxemia. Tippimanchai et al. (2018) suggested that DEX could reduce the number of alveolar macrophages and inhibit their phagocytosis in septic mice. García et al. (2003) suggest that α_2_-AR controls phagocytosis and chemotaxis in primary cultured rat peritoneal macrophages, maintaining phagocytosis at optimal levels.

All in all, the difference between the results of animal experiments and clinical trials deserves further study.

### 2.3 Adaptive Immune Responses

Adaptive immune responses refer to the whole process in which antigen-specific T/B lymphocytes are activated, proliferated and differentiated into effector cells to produce a series of biological effects.

#### 2.3.1 B Cells

B cells participate in the immune responses through a variety of ways: produce antibodies, differentiate into plasma cells, act as APCs, and secrete various cytokines to regulate the activities of other immune systems and immune cells. Under antibody-mediated autoimmune conditions, B cells play a key role in humoral immunity.

Whether α_2_-AR are expressed on the surface of B cells is unclear. When DEX is administrated with middle and high doses, it can inhibit the release of IL-1, IL-6, TNF-α and PGE2, increase the release of IL-2, and play an anti-nociceptive role in acute inflammatory visceral pain, thereby inhibiting visceral hypersensitivity (Ma et al., 2017; Liu et al., 2018). Also, DEX activates the B cell signaling pathway by inhibiting the p38 mitogen-activated protein kinase/nuclear factor K-light chain enhancer, increasing the serum IL-2 level of ovarian cancer rats and enhancing the immune function (Cai et al., 2017).

However, it was found that there was no significant difference in the number of B lymphocytes between the DEX group and the control group, suggesting that DEX had little effect on humoral immune response of patients undergoing oral cancer surgery (Huang et al., 2021). Wu R. S. et al. (2015) found that continuous intravenous infusion of DEX during general anesthesia can reduce the number of B cells, which effectively inhibits the perioperative stress response of children with brain tumors. The specific mechanism of the effect of DEX on humoral immune function is still unclear, and its effect on humoral immune function of patients with malignant tumor needs further study.

#### 2.3.2 T Cells

T cells are one of the main members of lymphocytes. They have many biological functions, including killing target cells directly, assisting or inhibiting B cells to produce antibodies, responding to specific antigens and producing cytokines. The immune response caused by T cells belongs to cellular immunity.

##### 2.3.2.1 The Amount of CD3^+^, CD4^+^, CD8^+^ and CD4+/CD8+ Ratio

CD3 molecule is a characteristic mark on the mature T cells and the number of it reflects the total number of T cells, and its increase indicates the enhancement of immune function. CD4^+^ T cells are auxiliary T cells, which play an accessory role in the induction of cellular and humoral immunity. CD8^+^ T cells are mainly immunosuppressive cells, which inhibit the function of other immune cells. The decrease of the CD4+/CD8+ ratio indicates poor immune function, and a large decrease often indicates the severity of the disease and poor prognosis (Huang et al., 2021).

Related studies have confirmed that compared with the control group, the amount of CD3^+^, CD4^+^ cells and the ratio of CD4+/CD8+ in the DEX group were significantly increased, while the percentage of CD8^+^ cells was significantly decreased (Wu R. S. et al. (2015); Huang et al., 2021; Wu R. S. et al. (2015); Yang et al., 2017; T. Zhao et al., 2013). Moreover, clinical trials found that intraoperative continuous intravenous infusion of DEX in adults can significantly improve cellular immune function (Liang et al., 2012). Clinical studies also show that DEX can regulate the perioperative immune response of patients undergoing radical surgery for breast cancer, colon cancer and gastric cancer, which is beneficial to enhance the immune function of cancer patients and promote postoperative recovery. And the incidence of gastrointestinal reaction and postoperative cognitive dysfunction in the DEX group are significantly lower than those in the control group (Wang et al., 2015; Yang et al., 2017; Wang and Li, 2018). Related studies believe that DEX can maintain better perioperative cellular immune function, reduce cellular immune suppression and hematogenous metastasis, and play a role in postoperative immune protection. As for cytokines, Yang et al. (2017) considered that the concentrations of IFN-γ, IL-2, IL-10 and IL-6 in DEX group were significantly increased.

##### 2.3.2.2 The Balance Among Th1, Th2, Th17 and Regulatory T Cells (Tregs)

The balance among T cell subsets is crucial for the homeostasis of the immune system and is a hot spot in current research (Zhao et al., 2013). The number of CD4^+^ T cells accounts for 65% in peripheral blood. The previous studies on CD4^+^ T cells are more detailed, and we will focus on this review. The Th0 cells is an initial CD4^+^ T cell unstimulated by antigen and can differentiate into Th1, Th2, Th17, and Treg cells under different cytokine environments.

Th1 mainly secretes IL-2 and IFN-γ, activates T lymphocytes and macrophages, mediates cellular immune response, and reduces postoperative infection (Lee et al., 2018). It is also believed that the increased secretion of IL-2 and TNF-α can activate inflammatory responses, promote leukocyte adhesion, and provide conditions for the further development of inflammation (Jeong et al., 2016; Elmoutaz Mahmoud and Rashwan, 2018). Th2 mainly secretes IL-4, IL-6 and IL-10, induces B lymphocytes to secrete immunoglobulin (Lee et al., 2018), promotes humoral or antibody mediated immunity, and suppresses cell-mediated immune responses (Kurosawa and Kato, 2008; Webster and Galley, 2009). At present, IFN-γ, IL-2 and IL-4 are the main indicators that reflect the balance of Th1 and Th2. However, the effect of DEX on the balance of T cell subsets (Th1/Th2) is controversial.

From the results of the meta-analysis, Wang Y. et al. (2019) belived that DEX increased the ratio of Th1/Th2. Besides, in patients undergoing laparoscopic cholecystectomy, the ratio of IFN -γ/IL-4 and Th1/Th2 cytokines in DEX group were higher than that in control group. In breast cancer patients, the levels of IL-2 and IFN-γ secreted by Th1 in DEX group increased, while the level of IL-4 secreted by Th2 did not change much, indicating that DEX can inhibit the transformation of Th1 to Th2 (Cardinale et al., 2011). DEX attenuates Th2 polarization, maintains a relatively stable balance of Th1/Th2, and reduces surgical stimulation and inflammatory response. Therefore, DEX can maintain the immune balance of the patient population and protect the cellular immune function of the patient (Wang et al., 2015). These findings suggest that DEX may increase the Th1/Th2 ratio, thus playing an immunomodulatory role in patients under stress from surgery and anesthesia (Song et al., 2014).

The above research believes that DEX can shift the balance to Th1, but the following research believes that DEX is more inclined to Th2. Inada et al. (2005) found that DEX reduced the Th1/Th2 ratio, leading to a shift towards Th2. DEX combined with spinal anesthesia could promote the mRNA expression and protein secretion of IL-4 and IL-10 in female patients and newborns after cesarean section, while the mRNA expression and protein secretion of TNF-α and IL-2 in the DEX combined with spinal anesthesia group were significantly lower than that in the control group. Although cesarean section may affect the levels of IL-2 and IL-4, but it turns out that there is no significant difference compared with normal delivery. Therefore, DEX combined with intraspinal anesthesia can reduce the adverse reactions of puerpera after cesarean section, and promote the transformation of Th1 cytokines to Th2 cytokines (Shi and Zhang, 2019). In the anesthesia of patients with colon cancer radical operation, the indexes of Th1 and Th1/Th2 in DEX group were lower than that in the control group, which promoted the transformation of Th1 cytokines to Th2 cytokines, and DEX can significantly inhibit the activation of NF-κB, soluble intercellular adhesion molecule-1 (sICAM-1), IL-8 and IL-6 caused by anesthesia (Wang and Li, 2018). However, it was also found that the ratio of Th1/Th2 in DEX group did not change significantly during anesthesia in healthy patients (Kallioinen et al., 2019).

Th17 cells are another recently discovered helper T cell that differentiates under the stimulation of IL-6 and IL-23. The Th17 secrete IL-17A, which has obvious pro-inflammatory effect (Song et al., 2014), and can enhance the recruitment of neutrophils in the inflammatory site (Zhang et al., 2018a). IL-17A is considered to coordinate the local immune response and host defense against foreign pathogens in the autoimmune system. Regulatory T cells (Tregs) are a subset of T cells that can inhibit the immune response of cancer patients, and closely related to the occurrence of autoimmune diseases. Their abnormal expression can lead to autoimmune diseases, in which IL-17 and IL-10 are the main indicators of Th17 and Tregs, respectively. Moreover, the Th17/Tregs ratio has been reported to play an important role in immune regulation (Park et al., 2005; Singh et al., 2007; Guo et al., 2010; ChangHee and Chen, 2011; Zhang et al., 2011; Gu et al., 2013).

Similarly, there are different opinions about the effect of DEX on the balance of Th17 and Tregs. In patients undergoing radical resection of colon cancer, the number of Tregs in the DEX group was higher than that in the control group, indicating that DEX can promote the transformation of primitive T cells into Tregs (Wang and Li, 2018). However, in laparoscopic cholecystectomy anesthesia, DEX is associated with the decrease of Tregs cytokines IL-4 and IL-10 and can dose-dependently modulate the inflammatory response (Lee et al., 2018). In the mouse model, Song et al. (2020) deemed that DEX could inhibit IL-17A storm induced by acute lung injury to a certain extent, and could significantly reduce the amount of proinflammatory cytokines in bronchoalveolar lavage fluid (BALF).

In general, DEX can regulate the balance of Th1, Th2, Th17 and Tregs, and it can not only inhibit inflammation, but also alleviate immunosuppression, so it has strong immunomodulatory effect and better clinical therapeutic effect (Song et al., 2020).

##### 2.3.2.3 Brief Summary

The possible reasons for DEX to improve cellular immunity are as follows: 1) DEX can selectively activate α_2_-AR in central and peripheral nervous system, reduce sympathetic activity and serum catecholamine concentration, and further alleviate surgical stress and its coupled immunosuppression (Xu and Wang, 2003); 2) DEX reduces perioperative opioid use and thus reduces the immunosuppressive effects of opioids (Kallioinen et al., 2019); 3) DEX has a certain analgesic effect, and application before surgical trauma stress can prevent the sensitization of the central and peripheral nerves, reduce the pain caused by traumatic stimulation, achieve preemptive analgesia, and enhance the body’s immune function (Wu R. S. et al., 2015); 4) DEX has anti-inflammatory and organ protective effects on ischemia and hypoxia injury, thereby maintaining body homeostasis and helping to improve immunity (Wang et al., 2015; Ma et al., 2017). In the future, we will need to study these potential mechanisms at the cellular and molecular levels, analyze immune cells, and detect the mRNA expression levels of transcription factors and chemokines (Huang et al., 2021).

**FIGURE 1:**
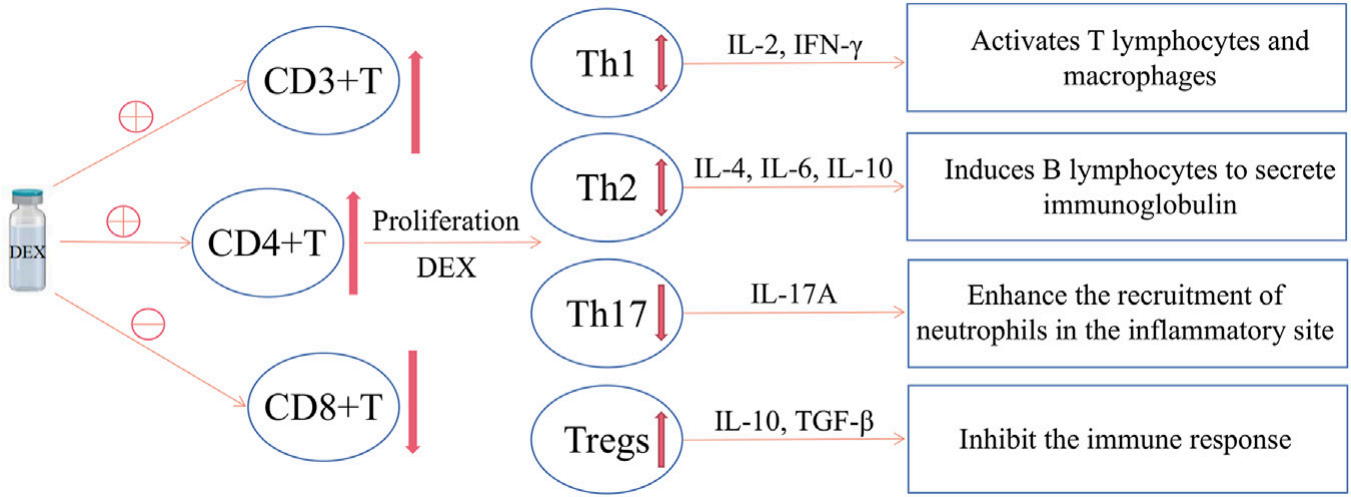
The effect of DEX on T cells. DEX can increase CD4^+^ T cells, decrease CD8^+^ T cells, and increase the CD4+/CD8+ value; meanwhile, DEX can promote the differentiation of CD4^+^ T cells into Tregs, reduce Th17 and the Th17/Tregs value; however, there is currently no consensus on the effect of DEX on Th1 and Th2.

In fact, T cells are mainly divided into T helper cells (CD4^+^ cells), cytotoxic T cells (CD8^+^ cells), memory T cells (CD4^+^ or CD8^+^ with antigenicity), Tregs, natural killer T cells (NKT cells) and δγ T cells (Chaplin, 2010). However, at present, studies mainly focus on the effect of DEX on the amount of CD3^+^, CD4 +, CD8^+^ and the ratio of CD4+/CD8+. The effects of DEX on memory T, NKT and δγ T cells are not involved at present, and there are few studies on molecular level, which may become the direction of future research. Figure 1 summarizes the effects of DEX on T cells.

### 2.4 DEX and Cancer

DEX can improve the coagulation and immune function of patients with colon cancer, and the CD4+/CD8+ of the DEX group after surgery is higher than that of the control group (Zhao and Li, 2020). DEX can reduce the immunosuppression of patients with oral cancer by increasing the percentage of CD3^+^, CD4^+^, DCs and CD4+/CD8+ ratio (Huang L et al., 2021). DEX can effectively inhibit the activation of IGF2 signal pathway, improve the immune function of ovarian cancer rats, inhibit the invasion and migration of ovarian cancer cells, and significantly increase the percentage of CD4^+^, CD8^+^ and the ratio of CD4+/CD8+ (Tian et al., 2019). The DEX infusion may improve the surgical outcomes of ovarian cancer by inhibiting the surgical stress response and the release of stress mediators (Shin et al., 2021). CD3^+^ T cells and CD4+/CD8+ in DEX group were significantly higher than that in the control group, which could reduce the perioperative inflammatory response and improve the cellular immune function of patients undergoing thoracoscopic radical resection of lung cancer (Kong and Lu, 2018; Zong et al., 2021). DEX may inhibit p38MAPK/NF-κB signaling pathway to enhance the immune function of ovarian cancer rats (Cai et al., 2017). DEX can alleviate the immunosuppression caused by circulatory fluctuation in patients with gastric cancer (Zheng et al., 2020). The levels of IFN-γ and IL-10 in the DEX group were lower than those in the control group, and the percentage of CD4+/CD8+ cells was higher than that in the control group, which reduced the perioperative stress response of rectal cancer patients and protected the cellular immune function (Zhang et al., 2018b). DEX can effectively alleviate the release of inflammatory factors in patients undergoing radical gastrectomy for gastric cancer, possibly by down-regulating the activation of NF-κB. In addition, DEX can also regulate the reduction of CD3^+^ and CD4^+^ subsets, so as to improve the impaired immune function (Dong et al., 2017). It also reduces the severity of early postoperative pain and opioid consumption in patients with uterine cancer (Cho et al., 2021). Continuous intravenous infusion of DEX during general anesthesia can effectively inhibit the perioperative stress response of children with brain tumors and reduce cellular immune suppression (Wu L et al., 2015). All in all, the current evidence shows that DEX can reduce immunosuppression in tumor patients, which may have certain significance in limiting tumor spread and invasion. Of course, the current studies has the problem of insufficient sample size, or most of the studies are based on animal experiments, so high-quality clinical randomized controlled studies in the future are necessary to demonstrate this conclusion.

### 2.5 DEX and Clonidine

Studies have shown that under acute inflammatory conditions, α_2_-AR agonist drugs can regulate the inflammatory process and immune pathways, of which receptor-mediated action is one of the important mechanisms (Flanders et al., 2019). Compared with clonidine, DEX has 8-fold higher affinity for α_2_-AR (Bhana et al., 2000). It was found that clonidine and DEX at relevant concentrations did not affect the chemotaxis, phagocytosis or superoxide production of human neutrophils. These findings indicate that when used in patients with infection, sepsis, or systemic inflammation, the type of α_2_-AR agonist is not the focus of attention (Nishina et al., 1999). A trial with no risk of bias compared DEX to clonidine found that target sedation was achieved in more patients treated with DEX and less need for additional sedation. Evidence on the use of clonidine in the intensive care unit (ICU) is very limited. DEX can effectively reduce the hospitalization time and extubation time of patients in ICU (Cruickshank et al., 2016). Unlike those reported in rodents, clonidine and DEX do not directly inhibit cytokine release from human lung macrophages (Piazza et al., 2016). In short, DEX and clonidine, which are also α_2_-AR, have very few comparative studies on their effects on immune cells, and this may be a future research direction.

## 3 Conclusion and Perspectives

The adrenergic signaling pathway has an immunomodulatory effect and has been extensively studied (Sharma and Farrar, 2020). As a highly selective agonist of α_2_-AR, DEX plays an important role in the inflammatory immune system, and this new direction has also triggered a series of studies on DEX in clinical diseases.

**FIGURE 2:**
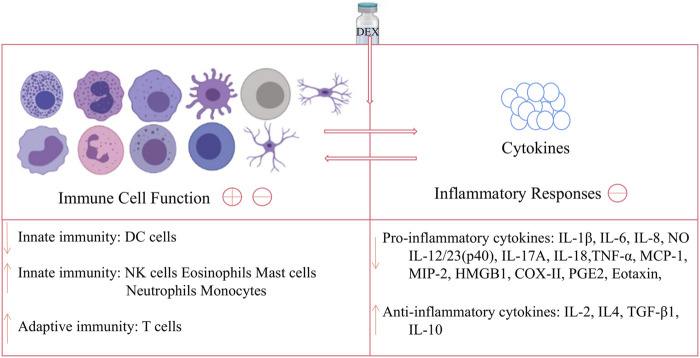
Effects of DEX on immune cell and inflammatory cytokines. DEX can act on DC cells to down-regulate innate immune function, while acting on NK cells, eosinophils, mast cells, neutrophils and monocytes to up-regulate this function; and DEX has no obvious effect on B cells, but can act on T cells upregulate adaptive immunity; meanwhile, DEX can down-regulate pro-inflammatory cytokines and up-regulate anti-inflammatory factors, thereby inhibiting the inflammatory response. Note: The image of the cells in the figure is from https://biorender.com/.

The article reviews the novel functions of DEX from the aspects of immune cells and related diseases. In general, DEX has double effects on innate immune response: on the one hand, it suppresses DCs function to play an immunosuppressive role; on the other hand, it promotes M2 polarization of macrophages, neutrophils clearance and enhance the amount of NKs to adjust immune function and play an anti-inflammatory role. In adaptive immune responses, DEX has little effect on the humoral response of B cells, but it can enhance cellular immunity by regulating the differentiation, number and proportion of T cell subtypes. And, the overall effect of DEX on immune cell function and inflammatory cytokines is shown in Figure 2. Furthermore, DEX can alleviate neuroinflammation and has a good therapeutic effect on autoimmune diseases, such as RA, osteoarthritis, tooth inflammation and colitis.

However, there are a lot of controversies at present. Some studies hold that DEX can inhibit the function of DCs *in vitro*, but some consider that it can increase the number of DCs in cancer patients to enhance immune function in clinical trials; besides, some think that DEX can increase the number of B cells, while others argue that it has little effect; and there are different opinions on the direction in which DEX modulates the balance of Th1/Th2 and Th17/Treg; similarly, there are different opinions on the mechanism of the new effect of DEX.

The reasons for the different results may be as follows: 1) due to the long-term use of DEX in some studies, the sensitivity of α_2_-AR may be down regulated, resulting in different results (Banati et al.,1993; Peng et al., 2013); 2) at present, the research on immune cells and inflammatory molecules is not thorough enough, and they may play different roles in different conditions; 3) the DEX may also play a role through other receptors and other mechanisms in different pathological conditions (Guo et al., 2018); 4) the dose was more than the clinical use *in vitro* studies; 5) studies on the effects of DEX on immunity and inflammation mostly focuses on monocytes/macrophages, microglia and T cells, and other immune cells are less involved, so it is normal for controversies to arise; 6) the research objects may be different, some are healthy, some are under the condition of illness; 7) it may be inaccurate to estimate the balance of T cells by measuring plasma cytokine concentration, because all IFN-γ and IL-4 in plasma are not only from Th1 and Th2 cells (Meng et al.,2020); 8) in addition, since most studies on the effect of DEX on immune responses are conducted in a clinical environment, patients have received various drug combinations, and some confounding factors may affect the results; 9) the time of sample collection and detection may be different, some may only detect the immediate reaction after medication, ignoring the results after 1–3 days (Lorton and Bellinger Denise, 2015).

The mechanism of DEX regulating immune cells and inflammatory mediators currently discovered includes: 1) DEX can active α_2_-AR on the immune cells membrane in the center and periphery, thereby regulating the expression of related inflammatory mediators; 2) DEX can directly or indirectly regulate the release of sympathetic neurotransmitters; 3) DEX has the direct anti-inflammatory effect, reduces the expression of pro-inflammatory mediators and increases the level of anti-inflammatory mediators; 4) DEX can promote natural sleep (Wu et al., 2016), which protects the body immune function, restores body energy and repairs the potential organ damage in the body (Besedovsky et al., 2012; Irwin and Opp, 2017; Joshua, 2021); 5) DEX can regulate M1/M2 polarization of macrophage and the balance among Th1, Th2, Th17 and Tregs; 6) DEX has a certain analgesic effect and helps to regulate the neuroendocrine immune network; 7) DEX reduces the release of pro-inflammatory factors through the TLR4-NF-κB-MAPK signaling pathway and the cholinergic anti-inflammatory pathway; 8) DEX exerts central anti-inflammatory effect by down regulating the expression of MCP-1; 9) DEX pretreatment can inhibit the expression of HMGB1, which is well known as a mediator of late inflammation, and it is also an early mediator of aseptic inflammation.

In the future, the role of DEX in different inflammatory and immune conditions, such as atherosclerosis and pulmonary infection, should be considered. DEX can significantly reduce the expression of MCP-1 (Wang et al., 2020), which plays an important role in the occurrence and development of RA, atherosclerosis, coronary heart disease and other inflammatory diseases (Deshmane et al., 2009; Xia and Sui, 2009). Also, DEX can enhance the expression of sirtuin-1 (SIRT1), which has been expected to become a new target for the treatment of cardiovascular diseases (Prola et al., 2017). Similarly, DEX can reduce the expression of VCAM-1 receptor integrin-4 (VLA-4) and lymphocyte associated molecule-1 (LFA-1), which is a good news for patients with atherosclerosis, long-term bedridden and undergoing major surgery. Also, DEX can up adjust PPARγ, which is the key to control the synthesis of pro-inflammatory cytokines, immunosuppression and cancer development (Wu et al., 2012). Moreover, DEX can down regulate HMGB1 in macrophages, which is an important inflammatory mediator in the late stage of sepsis and plays an important role in the pathogenesis of sepsis, tumor, arthritis and other inflammatory diseases (Andersson et al., 2018). Besides, DEX can enhance cellular immune response, so we can focus on its role in enhancing anti-tumor immunity.

All in all, we reviewed the anti-inflammatory and immunomodulatory mechanisms of DEX, comprehensively summarized the effects of DEX on immune cell function and inflammatory molecules, and also explained the role of α_2_-AR in the immune system, which provides a theoretical background for the application of DEX in immune-inflammatory related diseases.
